# Supplementary material for: Neurophysiological Mechanisms of Resilience as a Protective Factor in Patients with Internet Gaming Disorder: A Resting-State EEG Coherence Study
Source: J Clin Med. 2019 Jan 6;8(1):49. doi: 10.3390/jcm8010049 (PMC6352195; doi:10.3390/jcm8010049)
Supplement: Supplementary file 1 [file jcm-08-00049-s001.pdf]

Journal of
Clinical Medicine

Supplementary Materials: The following are available online at www.mdpi.com/xxx/s1, Table S1: Demographic and clinical characteristics between IGD and HC groups, Table S2: Pearson's correlation analysis between clinical features and EEG features.

Supplementary Tables

Table S1. The criteria for IGD in DSM-5

**Proposed Criteria**	
Persistent and recurrent use of the Internet to engage in games, often with other players, leading to clinically significant impairment or distress as indicated by five (or more) of the following in a 12-month period:	
1.	Preoccupation with Internet games.
2.	Withdrawal symptoms when Internet gaming is taken away.
3.	Tolerance
4.	Unsuccessful attempts to control the participation in Internet games.
5.	Loss of interests in previous hobbies and entertainment as a result of, and with the exception of, Internet games.
6.	Continued excessive use of Internet games despite knowledge of psychosocial problems.
7.	Has deceived family members, therapists, or others regarding the amount of Internet games.
8.	Use of Internet games to escape of relieve a negative mood (e.g., feelings of helplessness, guilt, anxiety).
9.	Has jeopardized or lost a significant relationship, job, or educational or career opportunity because of participation in Internet games.

IGD = Internet gaming disorder; DSM-5 = Diagnostic and Statistical Manual of Mental Disorders, Fifth Edition.

Table S2. Demographic and clinical characteristics between IGD and HC groups

	IGD (n = 35) Mean ± S.D	HC (n = 36) Mean ± S.D	F	P
**Demographic data**				
Age	23.94 ± 5.47	25.14 ± 3.60	1.190	.279
Education (years)	12.86 ± 1.70	14.75 ± 1.87	19.849***	< .001
Game usage in weekday (hour)	6.85 ± 3.94	0.88 ± 1.14	70.217***	< .001
Game usage in weekend (hour)	8.79 ± 3.88	1.06 ± 1.01	123.200***	< .001
**Clinical data**				
IAT	64.57 ± 15.37	29.11 ± 7.09	157.274***	< .001
CD-RISC	46.23 ± 21.68	74.14 ± 8.44	51.621***	< .001
BDI	18.57 ± 12.20	3.00 ± 2.99	55.304***	< .001
PWI	68.29 ± 30.34	28.86 ± 12.75	51.454***	<.001
IQ	107.31 ± 14.29	119.75 ± 10.45	17.586***	< .001

IGD = Internet gaming disorder; HC = healthy controls; S.D = standard deviation; IAT = Young's Internet addiction test; CD-RISC = Connor-Davidson resilience scale; BDI = Beck depression inventory-Ⅱ; PWI = Psychosocial Well-Being Index; IQ = intelligence quotient; P < 0.001***.

Table S3. Pearson's correlation analysis between clinical features and EEG features

**Overall (n= 71)**	**CD-RISC**	**BDI**	**PWI**
CD-RISC	1		
BDI	-0.793***	1	
PWI	-0.880***	0.888***	1
Alpha intrahemispheric coherence (all electrode pairs)	-0.166	0.278*	0.211
Alpha intrahemispheric coherence			
**IGD(n= 35)**	**CD-RISC**	**BDI**	**PWI**
CD-RISC	1		
BDI	-0.718***	1	
PWI	-0.864***	0.834***	1
Alpha intrahemispheric coherence (all electrode pairs)	-0.268	0.373*	0.311
Alpha intrahemispheric coherence (all electrode pairs)	-0.435**	0.417*	0.461**
Alpha interhemispheric coherence	-0.215	0.272	0.213
**HC (n= 36)**	**CD-RISC**	**BDI**	**PWI**
CD-RISC	1		
BDI	0.114	1	
PWI	-0.357*	0.611**	1
Alpha intrahemispheric coherence (all electrode pairs)	0.273	0.026	-0.118
Alpha intrahemispheric coherence (all electrode pairs)	0.440**	0.114	-0.151
Alpha interhemispheric coherence	0.149	-0.041	-0.202

CD-RISC = Connor-Davidson resilience scale; BDI = Beck Depression inventory-Ⅱ; PWI = Psychosocial Well-Being Index; IGD = Internet gaming disorder; HC = healthy controls; The mean value of each EEG variable was included; $P < 0.05$*, $P < 0.01$**, $P < 0.001$***.
